# P300‐mediated modulations in self–other processing under psychedelic psilocybin are related to connectedness and changed meaning: A window into the self–other overlap

**DOI:** 10.1002/hbm.25174

**Published:** 2020-08-21

**Authors:** Lukasz Smigielski, Michael Kometer, Milan Scheidegger, Cornelia Stress, Katrin H. Preller, Thomas Koenig, Franz X. Vollenweider

**Affiliations:** ^1^ Neuropsychopharmacology and Brain Imaging, Department of Psychiatry, Psychotherapy and Psychosomatics University Hospital of Psychiatry, University of Zurich Zurich Switzerland; ^2^ Translational Research Center University Hospital of Psychiatry, University of Bern Bern Switzerland

**Keywords:** anterior cingulate, connectedness, P300, psilocybin, psychedelic, self, self‐referential processing

## Abstract

The concept of self and self‐referential processing has a growing explanatory value in psychiatry and neuroscience, referring to the cognitive organization and perceptual differentiation of self‐stimuli in health and disease. Conditions in which selfhood loses its natural coherence offer a unique opportunity for elucidating the mechanisms underlying self‐disturbances. We assessed the psychoactive effects of psilocybin (230 μg/kg p.o.), a preferential 5‐HT1A/2A agonist known to induce shifts in self‐perception. Our placebo‐controlled, double‐blind, within‐subject crossover experiment (*n* = 17) implemented a verbal self‐monitoring task involving vocalizations and participant identification of real‐time auditory source‐ (self/other) and pitch‐modulating feedback. Subjective experience and task performance were analyzed, with time‐point‐by‐time‐point assumption‐free multivariate randomization statistics applied to the spatiotemporal dynamics of event‐related potentials. Psilocybin‐modulated self‐experience, interacted with source to affect task accuracy, and altered the late phase of self‐stimuli encoding by abolishing the distinctiveness of self‐ and other‐related electric field configurations during the P300 timeframe. This last effect was driven by current source density changes within the supragenual anterior cingulate and right insular cortex. The extent of the P300 effect was associated with the intensity of psilocybin‐induced feelings of unity and changed meaning of percepts. Modulations of late encoding and their underlying neural generators in self‐referential processing networks via 5‐HT signaling may be key for understanding self‐disorders. This mechanism may reflect a neural instantiation of altered self–other and relational meaning processing in a stimulus‐locked time domain. The study elucidates the neuropharmacological foundation of subjectivity, with implications for therapy, underscoring the concept of connectedness.

## Graphical abstract

Self‐referential processing pertains to the cognitive organization and perceptual differentiation of self‐stimuli, and its experimental perturbation offers a unique opportunity for elucidating the underlying mechanisms. We assessed the psychoactive effects of the potent preferential 5‐HT1A/2A agonist psilocybin during a verbal self‐monitoring task with assumption‐free multivariate randomization statistics applied to brain event‐related potentials. Treatment with psilocybin modulated self‐experience, interacted with self/other stimuli for task accuracy, and altered the electric field configurations during the P300 timeframe.

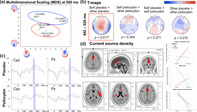

## 1 INTRODUCTION

Self refers to one's identity and the demarcated subject of experience. The concept has received notable interest across diverse research areas, ranging from philosophy (Metzinger, 2004; Zahavi, 2010) to psychiatry (Kircher & David, 2003) and neuroscience (Chen & Huang, 2017; Christoff, Cosmelli, Legrand, & Thompson, 2011). As recognizing “me” from “not me” is a fundamental principle of an intact sense of self (Decety & Sommerville, 2003), a key research focus has been the mechanisms underlying the differentiation between self‐ and other‐related stimuli. Neuroscientific studies using electroencephalography (EEG) and magnetoencephalography to index event‐locked cortical responses suggest that sequential (from early sensory to more semantic) brain processes are implicated in this differentiation (Walla, Duregger, Greiner, Thurner, & Ehrenberger, 2008). Previous experiments have indicated that the neural processing of self‐related stimuli differs from other‐related stimuli within as soon as 100–200 ms. The former tend to have lower amplitudes of the N1/N170 event‐related potential (ERP), while the latter tend to have higher amplitudes of the P2/N2 ERP (Caharel et al., 2002; Keyes, Brady, Reilly, & Foxe, 2010). These differences are critically engaged in the early detection of physical features (Krumbholz, Patterson, Seither‐Preisler, Lammertmann, & Lütkenhöner, 2003; Obleser, Scott, & Eulitz, 2006). This stage seems to reflect automatic sensory‐feedback mechanisms (Heinks‐Maldonado, Mathalon, Gray, & Ford, 2005) for detecting expected, prioritized, and overlearned input associated with the self (e.g., one's own voice). During a later processing phase of the P300 component (peaking within 300–600 ms), neural signatures from self‐related stimuli were also found to differ from other‐related stimuli (with typically higher amplitudes for the former), reflecting distinct activity in frontoparietal circuits (Knyazev, 2013) implicated in further elaborative processes and cognitive categorization (Polich, 2012). For example, a larger P300 amplitude was found for the sound of one's own name versus other words (Berlad & Pratt, 1995), self‐referent versus unrelated possessive pronouns (Zhou et al., 2010), and autobiographical memories versus other memories (Gray, Ambady, Lowenthal, & Deldin, 2004), suggesting a processing bias for stimuli pertaining to the self. Notably, a disruption of those two processing stages has been implicated in the pathophysiology of psychiatric symptoms, such as schizophrenia (Bühler et al., 2016; Qiu, Tang, Chan, Sun, & He, 2014), bipolar disorder (Zhao, Luo, et al., 2016; Zhao, Yao, et al., 2016), and depression (Tripathi, Mishra, Tripathi, & Gurnani, 2015).

Despite this progress in our understanding of the spatiotemporal brain dynamics of self–other differentiation, its neuropharmacological underpinnings, possible modulators, and corresponding subjective effects remain largely unknown. To further elucidate these questions, we administered the serotonergic agonist psilocybin (vs. placebo) to healthy human subjects and quantified their spatiotemporal brain activity using EEG in combination with topographic analysis of variance (TANOVA). Serotonin signaling and 5‐hydroxytryptamine 2A (5‐HT2A) receptors in particular might be implicated in self–other differentiation, as classic psychedelics, such as psilocybin (4‐phosphoryloxy‐*N*,*N*‐dimethyltryptamine, https://pdsp.unc.edu/pdspweb/), have been shown to induce marked alterations in the experience of self (Studerus, Kometer, Hasler, & Vollenweider, 2011). One salient aspect of psychedelic effects is so‐called ego‐dissolution, a state of perceived blurred demarcation between self and non‐self (Dittrich, 1998; Letheby & Gerrans, 2017; Preller & Vollenweider, 2016). Moreover, psilocybin was found to alter internally and externally induced neuronal activity (in association with drug‐attenuated alpha oscillations and a resulting imbalance of excitability in the absence or presence of an external stimulus) (Kometer, Schmidt, Jäncke, & Vollenweider, 2013) and also to modulate the N1/N170 (Bravermanová et al., 2018; Kometer et al., 2013; Kometer, Cahn, Andel, Carter, & Vollenweider, 2011; Umbricht et al., 2003) and P300 components (Bravermanová et al., 2018; Kometer et al., 2012) via 5‐HT2A receptor activation. Furthermore, altered delta oscillations, which are crucial for the P300 component (Güntekin & Başar, 2016), have been previously associated with aberrant self‐experience (Carhart‐Harris et al., 2016; Kometer, Pokorny, Seifritz, & Volleinweider, 2015). Given that these observed psilocybin‐induced modulations of neural mechanisms are thought to underlie self‐referential processing, it is conceivable that the ego‐dissolution effect is mediated by an altered self–other (i.e., source) differentiation, with corresponding differences in the spatiotemporal properties of the N100 or/and P300 ERPs. To test this hypothesis, we quantified the psychotropic effects of psilocybin in healthy subjects performing a self‐monitoring task involving speech production with feedback varying in source (self vs other) and pitch (introduced to increase response variation and task difficulty) on three different levels: experiential, behavioral, and neural. These assays included subjective drug effects, voice recognition and misattribution, and cortical responsiveness to self‐ and non‐self‐related stimuli, respectively.

## 2 MATERIALS AND METHODS

### 2.1 Experimental design

The study employed a double‐blind, placebo‐controlled, within‐subject design with a counterbalanced order of administration.

### 2.2 Participants

Seventeen healthy right‐handed individuals (9 males, mean age 25.1 ± 4.1 years, mean verbal IQ 104.9 ± 10.7) were included in the analysis (details on recruitment and exclusion/inclusion criteria as well as a study flow diagram are given in the Supplementary Material, pp. 2–3). Written consent was obtained from subjects before enrollment. The experiment was approved by the Cantonal Ethics Committee of Zurich, Switzerland.

### 2.3 Drug and dosing

Psilocybin (230 μg/kg body weight, mean body weight of subjects 71.5 ± 16.5 kg) and placebo (lactose) were administered in capsules of identical appearance on two experimental days separated by at least 2 weeks to minimize carry‐over effects. The use of psilocybin was authorized by the Swiss Federal Office for Public Health, Department of Pharmacology and Narcotics, Bern, Switzerland.

### 2.4 Psychometrics

Drug effects were measured by the Altered States of Consciousness rating scale (5D‐ASC) (Dittrich, 1998; Dittrich, Lamparter, & Maurer, 1999), using 11 previously empirically extracted scales (Studerus, Gamma, & Vollenweider, 2010). The present analysis specifically utilized three self‐related processing dimensions reflecting (a) alterations in self‐experience, that is, *feelings of unity*, (b) lessened bodily reference, that is, *disembodiment*, and (c) altered attribution of meaning, that is, *changed meaning of percepts*, as well as one additional aspect related to audio‐visual changes (i.e., *audio‐visual synesthesia*; multisensory perceptual experiences), which was considered based on the task modality. The changed meaning dimension was included because the attribution of personal relevance to external stimuli may convey an important aspect of self‐relatedness (Northoff & Hayes, 2011). In addition, our previous functional magnetic resonance imaging (fMRI) studies identified prominent acute changes in this domain caused by psychedelic drugs and related to self‐related processing (Preller et al., 2017; Preller et al., 2018).

### 2.5 Task and procedures

The experiment started 75 min post‐administration, corresponding to the beginning of the plateau of peak subjective effects produced by orally administered psilocybin (Hasler, Grimberg, Benz, Huber, & Vollenweider, 2004). Each trial was initiated with a visual cue “A,” after which subjects pronounced the sound [a:] for about 1 s and received real‐time audio feedback that varied between the participant's own unaltered voice, the participant's pitch‐shifted voice, an unaltered “other” voice, and a pitch‐shifted “other” voice. The pitch shifting was integrated into this self‐monitoring task to introduce sufficient variation and to increase task difficulty. After each trial, subjects indicated via key press whether they heard their own voice, the voice of someone else, or whether they were unsure. Importantly, they were instructed to choose the “self” response whenever they recognized their own voice, even when it was pitch‐shifted. The visual stimuli (i.e., the vocalization cue, rating board, and fixation cross) were presented in black text on a white screen. A centrally situated fixation cross was shown for 1.2 s between each trial. The inter‐trial interval was variable and dependent on the response reaction time. A reaction was required within 5 s after the prompt, and responses after this time were considered missing data. An internal trigger pulse marking the onset of vocalization was generated on the rising edge of the incoming audio signal and activated the experimental conditions. For the “other” condition, the participant's voice was substituted with a previously recorded sample of a male or female utterance of the sound [a:] (for male or female participants, respectively). The pitch‐shifting of two semitones downwards was implemented in real time for the unaltered speech and added ahead of time for the prerecorded other voice using the same algorithm. In order to dampen the external auditory feedback and mask bone conduction effects, a pink (1/*f*) background noise of 60 dB was constantly played over the earphones. The volume level was maintained at 87–93 dB. An external trigger pulse marking the onset of vocalization was sent through the parallel port to the EEG data collection system for analysis.

A total of 280 trials, with 70 randomly ordered trials per condition, were conducted. More details on the study procedures and the technical equipment are given in the Supplementary Material (pp. 4–5).

### 2.6 EEG recording and preprocessing

EEG was recorded with the BioSemi Active Two acquisition system using 64 Ag/AgCl scalp electrodes (BioSemi, Amsterdam, The Netherlands). Additional electrodes were placed on the outer canthus of each eye and supraorbitally and infraorbitally with respect to the left eye in order to record horizontal and vertical electrooculography. The recording sampling rate was 2048 Hz. The preprocessing was performed using BrainVision Analyzer 2.1 (Brain Products, Munich, Germany). The signal was down‐sampled after recording to 512 Hz and band‐pass‐filtered between 0.5 Hz (12 dB/octave slope) and 20 Hz (48 dB/octave slope). Eye movements were removed from the signal by extended infomax (independent component analysis) and channels with noise were interpolated by spherical splines. Data were segmented into epochs spanning 200 ms prestimulus to 800 ms poststimulus, and sweeps exceeding 80 ± μV and gradients exceeding 30 μV/ms were excluded from further analyses. The mean ± *SD* of accepted epochs per condition were as follows: for placebo, 67.7 ± 2.0 ms (self), 68.6 ± 1.3 ms (self pitch‐shifted), 68.9 ± 1.3 ms (other), and 68.9 ± 1.4 ms (other pitch‐shifted); for psilocybin, 65.6 ± 3.7 ms, 66.8 ± 3.5 ms, 67.4 ± 3.1 ms, and 66.8 ± 4.5 ms (respectively). Finally, the epochs were averaged and baseline corrected from −200 to 0 ms. Data were also inspected visually to confirm the accuracy of automated procedures. The average reference was used for analysis. Data were analyzed from 61 channels, forming a uniform spherical head model (without P10, P9, or Iz).

### 2.7 Analysis of subjective drug effects and behavioral responses

A multinomial logistic regression was used to estimate the probability of correct, incorrect, and unsure responses in the behavioral task dependent on the drug treatment and experimental condition of source (“self”/“other”) and pitch (“unaltered”/“pitch‐shifted”). As the marginal count of missing responses was particularly low (0.17% and 0.99% of all responses for the placebo and psilocybin groups, respectively), these responses were not considered in the analysis. Two models were used to analyze the data: Model 1 was fitted with all the data, and Model 2 was fitted only with data based on unaltered voices (i.e., without pitch conditions), as our key interest was the genuine self–other contrast. The hypothesis assumed that the acute treatment with psilocybin, compared to placebo, would cause a decline in task accuracy manifested as an interaction with source, by blurring differentiation between the “self” and “other” stimuli. The significance level was set at *p* < .05.

### 2.8 General analysis approach to EEG data

Quantitative statistical inferences about neural events and their generators under experimental conditions were conducted using randomization statistics for the whole‐brain signal without assuming any a priori models, such as a particular set of channels or specific time window. The approach combined computational methods, as implemented in the MATLAB‐based Randomization Graphical User Interface (RAGU) software‐package (Habermann, Weusmann, Stein, & Koenig, 2018; Koenig, Kottlow, Stein, & Melie‐García, 2011), which is designed to analyze multichannel ERP data using randomization statistics. The analysis was conducted for the main conditions of interest, that is, treatment (psilocybin/placebo) and source (self/other; without pitch alterations, as we were specifically interested in the genuine source effects), as factors. For the randomization tests (topographic consistency test [TCT], TANOVA, and topographic analysis of covariance (TANCOVA)], 5,000 permutations (Manly, 2006) were performed over the whole 800‐ms poststimulus window, with a significance threshold of *p* < .05. While evaluating time windows, to account for temporal autocorrelation and multiple comparisons, the global duration statistics, as part of the RAGU software, were applied, using 5,000 randomized permutations with an alpha level of *p* = .05 (i.e., accepting periods longer than 0.95 of all randomly obtained effect durations) (Habermann et al., 2018).

### 2.9 Analysis of topographic configuration

In the first step, a TCT based on global field power (GFP) was used to evaluate the stability of voltage configurations, providing support (or a lack of support) for consistent neuronal sources (Koenig & Melie‐García, 2010). The test compares the GFP of an ERP for each time point with the GFP distribution at the same time point by randomly shuffling the data for each individual voltage value. The GFP is a quantifier of field strength regardless of topographic modulations, calculated as the root of the mean of the squared potential differences across all sensors $\left(\mathrm{GFP}=\surd \left[\sum_{i=1}^{N}\left(v_{i}-{\hat{v}}_{i}\right)\right]*2N\right)$ (Lehmann & Skrandies, 1980). The derived Monte Carlo *p*‐value reflects the level of probability with which the given configuration could have been generated by chance. Subsequently, data were subjected to a TANOVA (Strik, Fallgatter, Brandeis, & Pascual‐Marqui, 1998). This is a nonparametric randomization procedure that calculates global dissimilarities between experimental factors in electric scalp fields in the ERP grand means for each sampling point and tests the probability of these dissimilarities occurring under the null‐hypothesis. Since the difference in spatial distribution implies at least partially varying functional mechanisms, the test may allow for their disentanglement (Koenig et al., 2011). The two‐factorial design used a randomization procedure where each factor (treatment, source) was permuted separately. Because a fully free randomization may decrease the sensitivity for detecting an effect of a weaker factor (Koenig et al., 2011), the permutations were constrained such that each factor competes only against the permutations of itself and not against those of the other factor. Significant TANOVA effects were followed by construction of post hoc *t*‐maps and a multidimensional scaling method to further explore the spatial distribution of differences.

### 2.10 Link between neural data and subjective drug effects

A TANCOVA (Koenig et al., 2011) was conducted to test whether and in which time window and scalp configuration a behavioral continuous variable co‐varied linearly, using bootstrapping and randomization statistics. The method considers ERPs to be superimposed on a topographic map whose contribution to the ERP is proportional to external variables. This analysis was used to determine whether there was a consistent set of sources being activated in proportion to the drug effects. The calculation was performed on the difference in L2‐normalized ERPs between both “self” placebo and psilocybin and “other” placebo and psilocybin conditions as well as the difference in scores between placebo and psilocybin treatment, as measured by the ASC scale.

### 2.11 Source estimations

As differences in scalp topographic configurations must result from somewhat dissociable underlying neural generators, standardized low‐resolution brain electromagnetic tomography (sLORETA) (Pascual‐Marqui, 2002), a well‐established source localization method, was used to approximate putative brain areas within the drug by source interaction time window as determined by TANOVA. A paired test with 5,000 permutations was used to examine whether (*A*_1_–*A*_2_) = (*B*_1_–*B*_2_), where *A*_1_ and *A*_2_ are log‐transformed self and other values, respectively, for the placebo treatment, and *B*_1_ and *B*_2_ are self and other values, respectively, for the psilocybin treatment. sLORETA is an inverse solution approach for computing the smoothest of the possible three‐dimensional intracerebral distributions of the current source density (CSD), based on the MNI152 template and 6,239 voxels (5 × 5 × 5 mm) covering the gray matter. The regularization parameter (signal‐to‐noise ratio) was set to 100, but other values gave very similar results. The results were assessed with a two‐tailed *t*‐test at a *p* < .05 threshold.

## 3 RESULTS

### 3.1 Subjective effects of psilocybin

**FIGURE 1:**
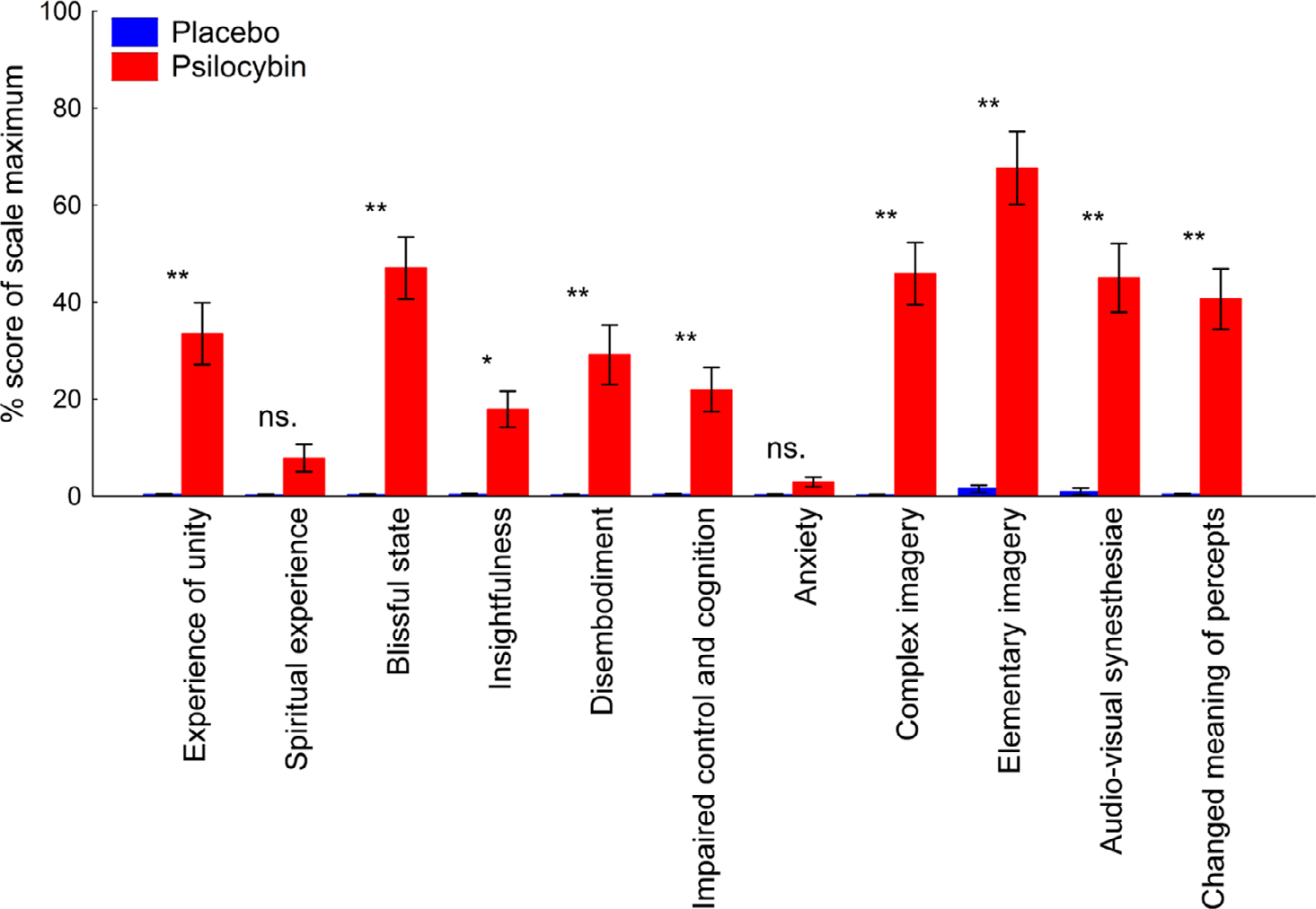
Subjective effects of psilocybin as measured along 11 dimensions of the Altered States of Consciousness rating scale (5D‐ASC). Scores are percentages of the scale maximum. Values are means and *SEs* with significance levels of Tukey's HSD tests as shown: **, *p* < .0001; *, *p* < .05; ns., not significant

First, we analyzed the subjective effects of placebo versus psilocybin treatment. Psilocybin‐modulated 5D‐ASC scores, as indicated by the significant interaction between drug and scale (*F*_10,160_ = 16.71, *p* < .0001), as well as main effects of scale (*F*_10,160_ = 17.29, *p* < .0001) and treatment (*F*_1,16_ = 83.05, *p* < .0001). With the exception of anxiety (*p* = 0.999) and spiritual experience subscale scores (*p* = .991), post hoc Tukey's HSD tests revealed a significant increase for all other subscale scores after psilocybin administration compared to placebo (for insightfulness, *p* = .024; for all others, *p* < .0001) (Figure 1).

### 3.2 Self‐monitoring task performance

**FIGURE 2:**
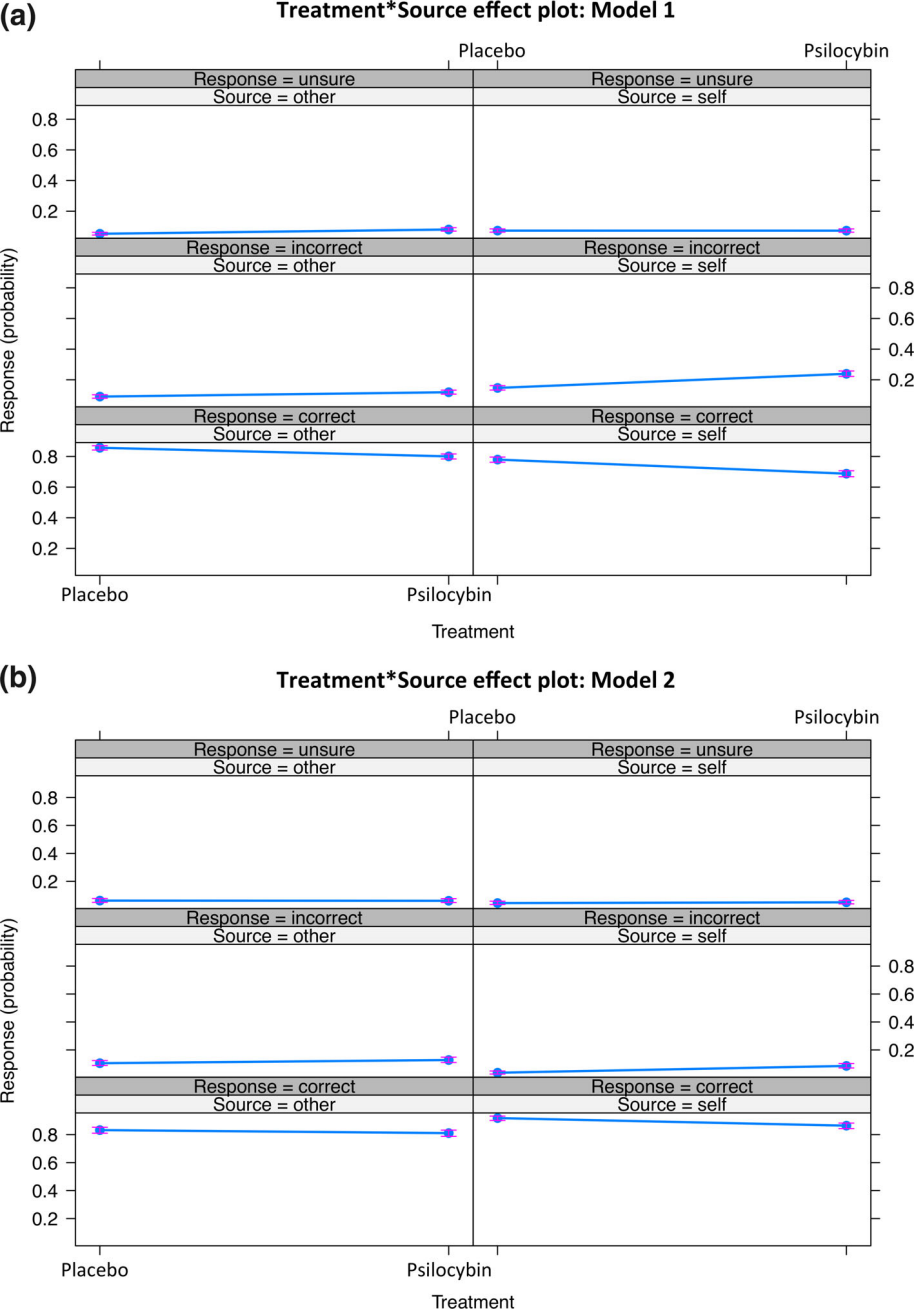
Response probability visualized as treatment by source interaction effects in the self‐monitoring task. Three formats of responses (correct, incorrect, unsure) are depicted on the plot as dependent on the source (“self,” “other”) and treatment (placebo, psilocybin). Model 1 (a) includes all data, and Model 2 (b) discards the pitch‐shifted conditions

We also investigated the impact of treatment with psilocybin on behavioral responses in the task, testing the key hypothesis of the significance of a treatment by source interaction. The number of correct, incorrect, and unsure responses for the placebo group were 3,823 (80.43%), 628 (13.21%), and 302 (6.35%) versus 3,442 (73.03%), 911 (19.33%), and 360 (7.64%) for the psilocybin group, respectively. The results of the multinomial logistic regression are reported in Table 1, showing which predictors have a significant effect on the response. Conforming with our hypothesis, the positive coefficients and significant *p*‐values for Models 1 and 2 (*β* = .373, *p* = .004; *β* = .676, *p* = .003) suggest the probability of responding inaccurately increases under psilocybin treatment. This interaction effect is plotted in Figure 2, indicating that drug had a bigger effect on correct and incorrect responses when the source voice was “self” than when the source was “other,” as represented by the difference in line slopes. The drug itself increased the probability of false recognitions in the model including pitch (*β* = .330, *p* = .004), but this effect became nonsignificant when pitch was discarded from the analysis (*β* = .223, *p* = .084). Drug alone had no significant effect on unsure responses. Separately, an interaction of treatment, source, and pitch was tested by including a triple interaction term in the model, yielding nonsignificant results (*β* = .264 and *p* = .085, for incorrect and unsure responses).

**TABLE 1:** Parameter estimates of the multinomial logistic regression analysis in the self‐monitoring task (*z*‐test of coefficients)

Predictor	Model 1				Model 2			
	Estimate	*SE*	*z*	Pr(>|*z*|)	Estimate	*SE*	*z*	Pr(>*|z*|)
Incorrect (intercept)	−2.127	0.090	−23.520	<2.2e‐16***	−2.069	0.095	−21.801	<2.20e‐16***
Incorrect: Treatment drug	0.330	0.114	2.890	0.004**	0.223	0.129	1.726	0.084
Incorrect: Source self	−0.955	0.134	−7.136	<9.59e‐13***	−1.141	0.181	−6.316	<2.694e‐10***
Incorrect: Pitch‐shift	0.039	0.118	0.332	0.740				
Incorrect: Treatment drug × source self	0.373	0.128	2.912	0.004**	0.676	0.226	2.989	0.003**
Incorrect: Source self × pitch‐shift	2.252	0.134	16.837	<2.2e‐16***				
Incorrect: Treatment drug × pitch‐shift	−0.012	0.135	−0.091	0.928				
Unsure (intercept)	−2.717	0.118	−23.096	<2.2e‐16***	−2.594	0.121	−21.522	<2.2e‐16***
Unsure: Treatment drug	0.245	0.148	1.654	0.098	0.006	0.172	0.038	0.970
Unsure: Source self	−0.159	0.154	−1.035	0.301	−0.431	0.185	−2.327	0.020*
Unsure: Pitch‐shift	−0.072	0.154	−0.468	0.640				
Unsure: Treatment drug × source self	−0.371	0.167	−2.220	0.026*	0.169	0.259	0.652	0.514
Unsure: Treatment drug × pitch‐shift	0.444	0.170	2.616	0.009**				
Unsure: Source self × pitch‐shift	1.074	0.170	6.337	2.34e‐10***				

*Note:* The *z*‐statistic reflects the ratio of the coefficient to the *SE* of the predictor, while Pr > |*z*| is the corresponding *p*‐value. The reference level was set to correct responses. Model 1 includes the whole data set, while Model 2 includes only the non‐pitch‐shifted conditions.

^***^ *p* <.0001.

^**^ *p* < .001.

^*^ *p* < .05.

### 3.3 Analysis of scalp topographies

**FIGURE 3:**
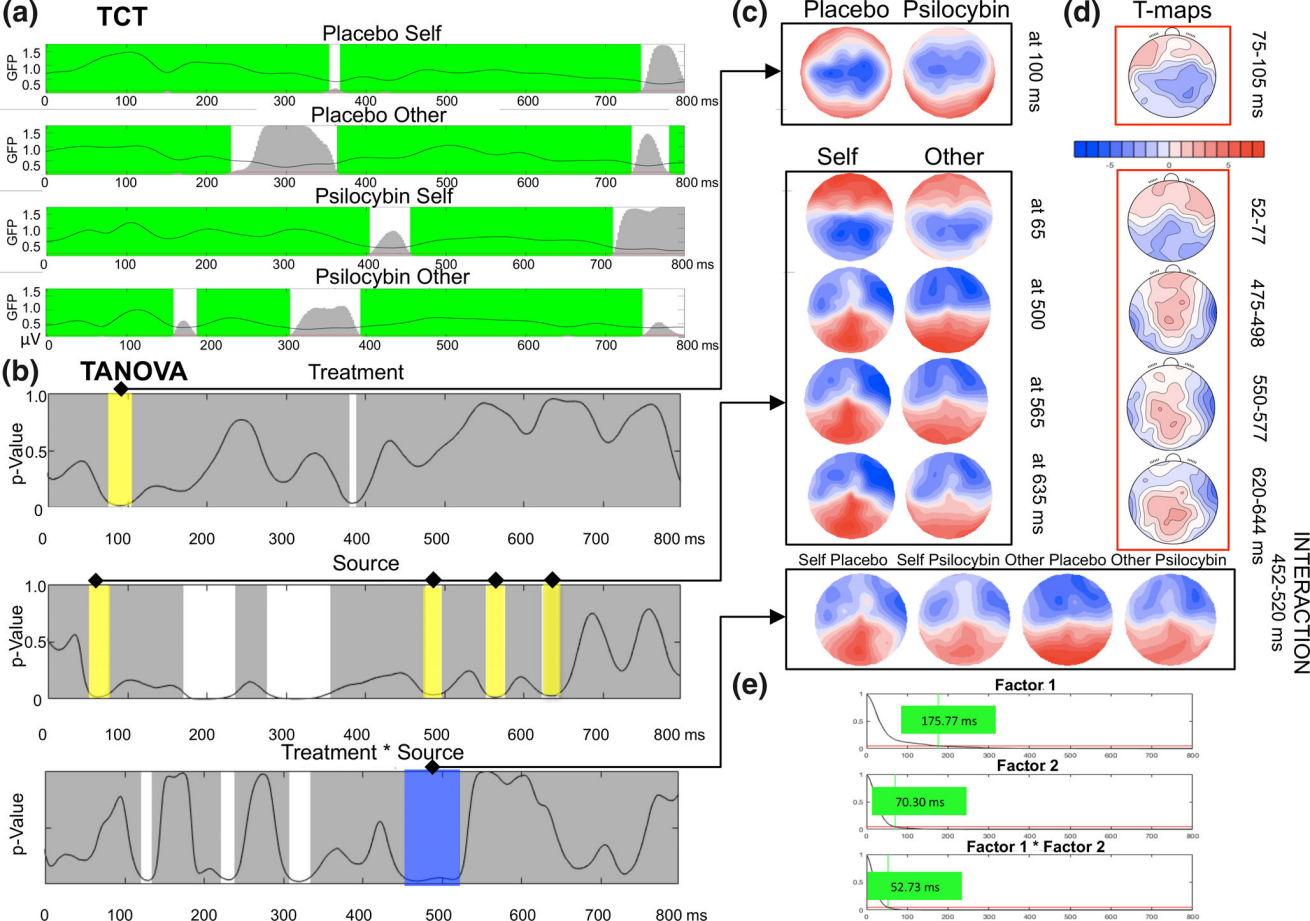
Topographic comparison for the whole epoch (0–800 ms) for treatment (placebo/psilocybin) and source (self/other). (a) Results of the topographic consistency test (TCT) for all four conditions with time windows in green marking significant effects (only these were considered for further analyses). The upper black curve represents the global field power (GFP) amplitude (in μV), the lower vertical line corresponds to the *p*‐value of .05, and the height of gray areas indicates the TCT *p*‐value. (b) Timeframes in white depict periods over which the null hypothesis probability *p* in the topographic analysis of variance (TANOVA) (*y*‐axis) as a function of time (*x*‐axis) was below .05; timeframes in yellow were during periods of consistent scalp configurations (those meeting the a priori criterion of duration <20 ms, to account for autocorrelation); the timeframe in blue meets the data‐driven criterion of global duration statistics. (c) Topographies of main effects and interactions. The arrows link the found time windows with the corresponding topographic maps. (d) *T*‐maps contrasting significant main effects (placebo > psilocybin, self > other). The interaction effect is depicted further in detail in Figure 4. (e) Global duration statistics for the main effects of Factor 1 (treatment), Factor 2 (source), and their interaction. Only the interaction time window (452–520 ms) met the duration statistics criterion (>52.73 ms)

Before performing TANOVA, we investigated the consistency of scalp electroencephalogram maps. The TCT (Figure 3a) indicated consistent activation for most of the 800‐ms period apart from the following intervals: 362–370 ms, 740–800 ms (placebo self), 231–363 ms, 731–780 ms (placebo other), 401–452 ms, 709–800 ms (psilocybin self), 157–186 ms, 301–394 ms, and 746–800 ms (psilocybin other). Since there was no evidence that the experimental conditions elicited verifiable scalp configurations, effects within these timeframes were not considered for further analyses.

**FIGURE 4:**
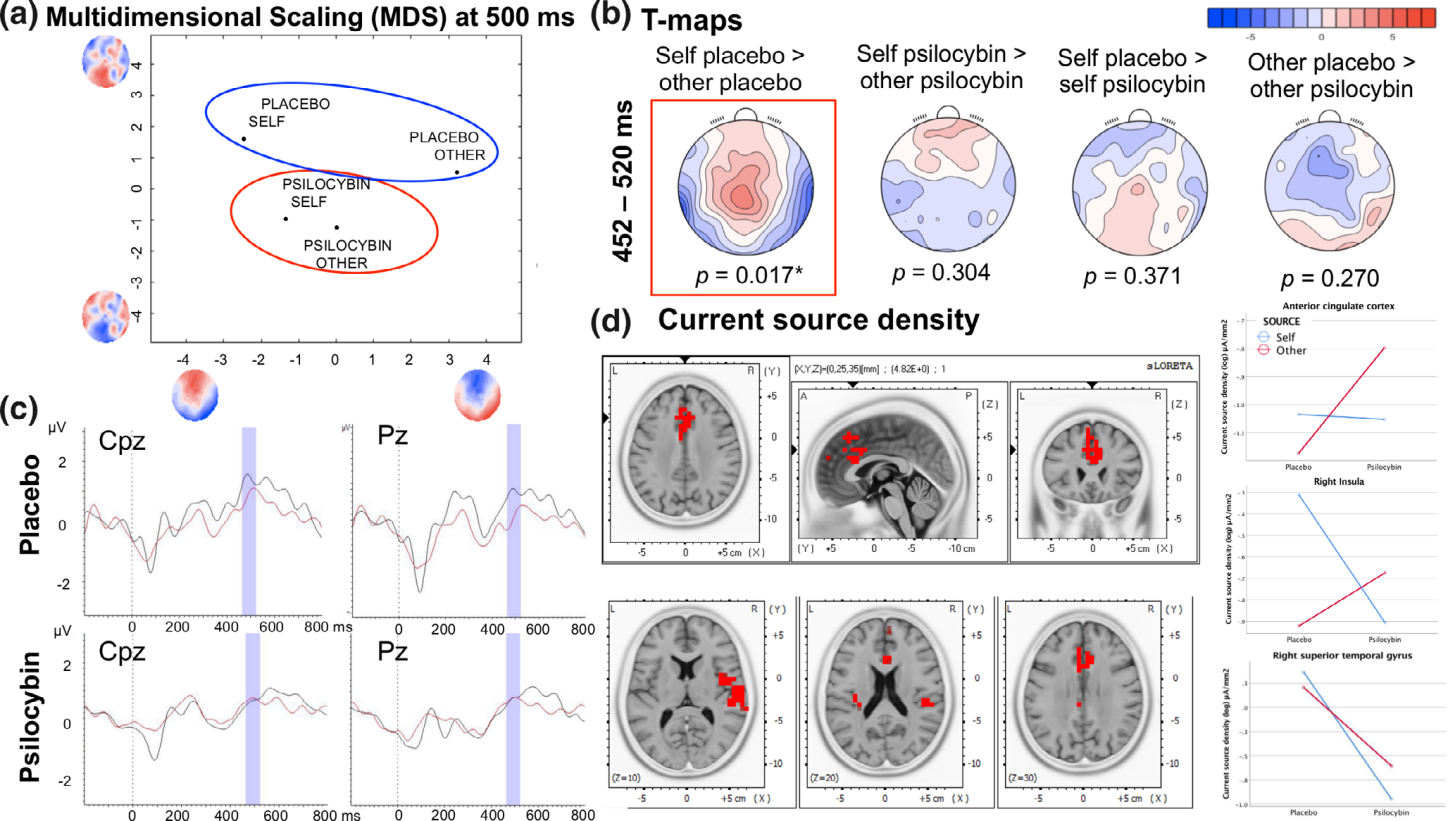
Detailed exploration of the topographic analysis of variance (TANOVA) interaction effects (452–520 ms). (a) The interaction found by the TANOVA was input into a multidimensional scaling (MDS) analysis by submitting all mean maps to a spatial principal component analysis (PCA). The spatial distribution of the eigenvector is represented by topographies along the *x*‐ and *y*‐axes. The MDS indicated that the drug by source interaction is primarily associated with a high difference between self and other for the placebo treatment, which is much smaller than that for the psilocybin treatment. (b) Post hoc *t*‐maps of interaction contrasts. Note that only the *t*‐map for self > other for the placebo treatment is significant (with maximal positivity in Cpz), while no significant difference was evident for self > other for the psilocybin treatment nor the other contrasts. (c) Illustration of waveforms recorded from Cpz (detecting maximal positivity in the aforementioned contrast) and Pz (cardinal neighboring sensor) electrodes (black, self; red, other; violet, 452–520 ms). (d) Estimates of current source densities, by calculating voxel‐by‐voxel paired self–other differences using standardized low‐resolution electromagnetic tomography (sLORETA). Significant source density differences are depicted in red (*p* = .012, corrected for multiple comparisons by the Nichols and Holmes method). Suprathreshold voxels were found primarily in the supragenual anterior cingulate (*t*_max_ = 4.82) and right insula (*t*_max_ = 4.06), as well as in the right superior temporal gyrus (*t*_max_ = 4.30). Maximal source density estimations are marked with arrows. The plots depict the current source density (CSD) values exported from the three main clusters

The TANOVA results, corresponding topographic maps, and *t*‐maps are depicted in Figure 3b–d, respectively. The TANOVA revealed a significant main effect of drug from 75 to 105 ms postvocalization onset. The corresponding *t*‐map in this timeframe was characterized by left anterior positivity (*t*_max_ at FT7 = 2.406) and right posterior negativity (*t*_min_ at P4 = −3.090). A significant main effect of source was found very early, between 52 and 77 ms, with *t*‐maps of almost symmetric frontal positivity and negativity propagating posteriorly (*t*_max_ at FT8 = 2.630, *t*_min_ at PO4 = −2.743). Late components included the 475–498 ms (*t*_max_ at FC2 = 3.079, *t*_min_ at T8 = −6.389), 550–577 ms (*t*_max_ at C1 = 3.034, *t*_min_ at T8 = −5.047), and 620–644 ms (*t*_max_ at CP2 = 3.332, *t*_min_ at FT8 = −5.100) time windows, with a characteristic positivity moving around the midline anterior to posterior areas, together with coexistent bilateral parietal negativities. A significant drug by source interaction was identified for the time window between 452 and 520 ms, which was also the longest period of significance. The interaction revealed when the effect occurred but did not determine which conditions accounted for it. Figure 4a–d presents further exploration of the interaction effects. Importantly, a series of post hoc *t*‐map contrasts (Figure 4b) provided more insight into this effect by showing that the scalp configurations between “self” and “other” differed significantly for placebo (*p* = .017, *t*_max_ at Cpz = 4.580, *t*_min_ at TP7 = −5.728), but not for psilocybin or other contrasts (*p* > .270; uncorrected *p* across four comparisons).

### 3.4 Current source densities

In order to discover more about the actual neural generators underlying the interaction effect, we interrogated this question using whole scalp data in the inverse space within the identified time window (452–520 ms). This showed significant modulations in CSD under psilocybin for *k* = 249 voxels (5 × 5 × 5 mm) localized primarily in the supragenual anterior cingulate (*x* = 0, *y* = 25, *z* = 35; *x* = 0, *y* = 25, *z* = 25), indicating a decrease for the “other” condition under psilocybin. Another concentrated cluster of suprathreshold voxels was found in the right insula (*x* = 50, *y* = −30, *z* = 20), with increases in CSD for the “self” condition under psilocybin. There was also an effect localized in the right superior temporal lobe (*x* = 65, *y* = −20, *z* = 10), in the right primary auditory cortex (Brodmann area 42), indicative of attenuated activity for psilocybin. The significant suprathreshold clusters from this analysis are depicted in Figure 4d, along with plots showing the extracted values according to treatment, source, and brain area.

### 3.5 Link between neurophysiology and subjective effects of psilocybin

**FIGURE 5:**
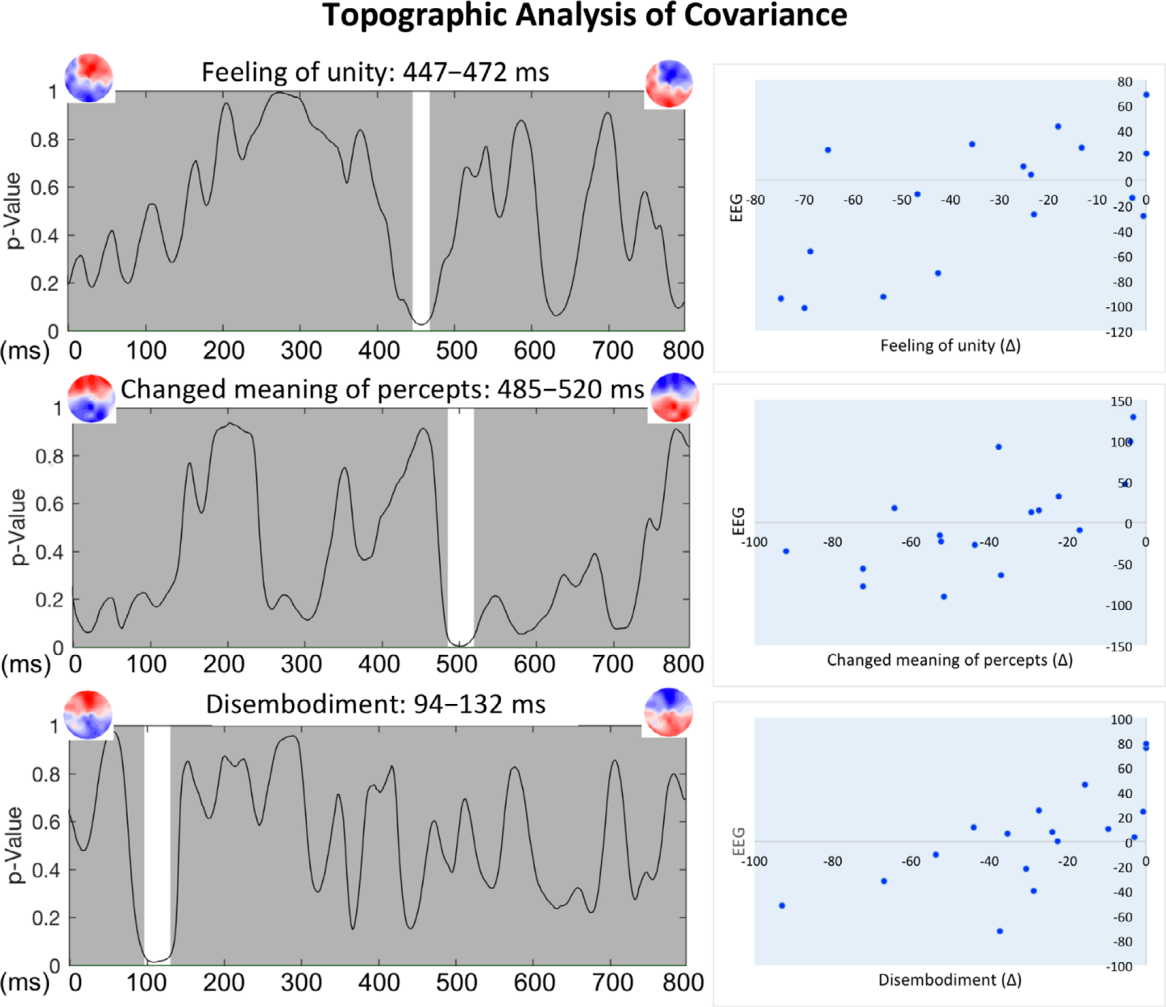
Results of a topographic analysis of covariance (TANCOVA). Five thousand iterations per time point were calculated for the placebo–psilocybin difference for self conditions and for subjective effects from the Altered States of Consciousness rating scale (5D‐ASC). White fields under the curve indicate periods over which the null hypothesis probability *p* was below .05. The regression plots on the right illustrate the individual strength of the covariance map with scale values along the *y*‐axis as arbitrary electroencephalography (EEG) units

Finally, we investigated the association between scalp signal and drug effects. The TANCOVA of the difference between “self” conditions revealed significant effects for the feeling of unity (447–472 ms), changed meaning of percepts (485–520 ms), and disembodiment (94–130 ms) (Figure 5). These significant associations (*p* < .05) occurred over intervals exceeding 20 ms, which we considered reliable and sufficiently accounting for temporal autocorrelation in this permutation‐based analysis (Guthrie & Buchwald, 1991), as applied in previous studies applying this method (Lancheros, Jouen, & Laganaro, 2020; Liverani et al., 2015). The initial 5 ms for the effect on the feeling of unity occurred in a period of inconsistent topographies based on the TCT. None of the “other” conditions were significant, apart from disembodiment in the time window between 335 and 370 ms, which was discarded because of inconsistent topographies.

## 4 DISCUSSION

The present human study, using a placebo‐controlled, within‐subject design, examined the effects of a potent psychedelic, psilocybin, on self–other distinctions during a verbal self‐monitoring task. First, we measured the self‐reported acute changes in consciousness and perception, and subsequently, we assessed the behavioral task responses. Furthermore, we investigated the whole scalp ERPs from the task, as well as the relationship between electrophysiology and subjective drug effects. As the key finding, the spatiotemporal analysis of the EEG signal during the whole epoch (0–800 ms) revealed that psilocybin lessened distinct self‐ and other‐related neural scalp configurations during the P300 timeframe (452–520 ms) compared to placebo. This interaction effect involved modulations of CSD in brain regions typically associated with self‐processes, including the anterior cingulate and insular cortex. Moreover, neural alterations in self‐processing induced by psilocybin co‐varied with drug‐induced subjective effects (including feeling of unity and changed meaning) within the same P300 time window. These results support the association of these neuronal mechanisms with psilocybin‐induced alterations in self‐experience.

### 4.1 Behavioral responses

The analysis of the judgment‐based behavioral ratings in the self‐monitoring task indicated an interaction effect between the action of psilocybin and the “self” and “other” categories on speech attribution accuracy. Specifically and critically, under the pharmacological stimulation, the probability of attributing the “self” voice to the “self” source dropped significantly more than the probability of recognizing the “other” voice as “other.” This finding confirms the hypothesis being tested and guiding the present work, which posits an alteration in self‐referential processing caused by the drug. Reports of psilocybin‐induced aberrant self‐experience, lessened ego‐centricity, or even states of “selflessness,” particularly under higher doses can be found in recent research literature (Smigielski, Kometer, et al., 2019; Smigielski, Scheidegger, Kometer, & Vollenweider, 2019). Impaired self‐monitoring for speech production and recognition suggests affected mechanisms of cognitive control and motor‐to‐sensory transformations (Nozari & Novick, 2017), but it may also reflect a unique profile of psychedelic compounds altering self‐related processes. Interestingly, a proneness to misattribute speech or externalization biases during self‐monitoring tasks has been linked to delusions and hallucinations in disease (Allen et al., 2004; Allen, Freeman, Johns, & McGuire, 2006), as is often induced (although more in the form of pseudo‐hallucinations) by psychedelic drugs such as psilocybin (Studerus et al., 2011).

### 4.2 Neural responses: Topographical configurations

In agreement with the behavioral responses from the self‐monitoring task, there was also a significant drug by source interaction for the neural data during the P300 processing stage. Post hoc *t*‐maps revealed a significant self–other difference in topographical configurations under placebo, while no such differences occurred under psilocybin (Figure 4b). Analogously, multidimensional scaling, which enables the visualization of similarities among datasets as Euclidean distances in low‐dimensional space, showed that self‐ and other‐related processing under psilocybin was associated with much more similar underlying neural events compared to placebo (Figure 4a). Additionally, visual inspection of waveforms for canonical P300 electrodes indicated overlaps in the significant time window (Figure 4c), suggesting a blurred differentiation between percepts of both self and other. This finding indicates that psilocybin alters self–other differentiation primarily by dissolving the neuronal differences between self and other during late (P300) processing stages.

Using TANCOVA, significant effects were observed for changed meaning of percepts and feeling of unity during the P300 timeframe (Figure 5); the former reflects a proneness to attribute changed meaning to perceptual events, while the latter suggests undifferentiated cognition between self and non‐self/other. We detected changes that coincided and covaried with the magnitude of subjective drug‐induced effects within the same timeframe, which further strengthens the evidence for psilocybin altering ordinary self‐experience. This comports with previous fMRI results showing the psychedelic substance LSD altered self‐ and other‐initiated social interactions and was associated with changes in meaning attribution (Preller et al., 2017; Preller et al., 2018). Based on these and other findings, we believe that meaning attribution is intrinsically tied to self‐processes through self‐relatedness, reward effects, and the valuation systems of organisms (de Greck et al., 2008; Northoff & Hayes, 2011). More broadly, altered meaning processing may be a key mechanism stimulating the putative therapeutic action of psychedelic compounds (Hartogsohn, 2018). Here, we specifically identified P300 as the time window associated with psilocybin‐induced subjective effects on the self, also supporting the established role of P300 in self‐referential processing (Knyazev, 2013).

### 4.3 Neural responses: CSDs

To further explore the spatiotemporal dynamics of the drug by source interaction, source localization was conducted during the detected P300 timeframe, revealing the underlying modulations of CSD in the supragenual anterior cingulate, right insular cortex, and right auditory cortex. Previous studies suggest that once represented in the orbitomedial prefrontal cortex, self‐referential stimuli are processed in the supragenual anterior cingulate, which subserves monitoring functions (Northoff & Bermpohl, 2004). Higher activity in this region was found for self‐referential compared to non‐self‐referential stimuli (Frith & Frith, 1999), irrespective of sensory modality or task (Northoff & Bermpohl, 2004). Accumulated evidence supports a pivotal role of cortical midline structures as the neural basis of the self (Qin & Northoff, 2011). In our study, for psilocybin, CSD was lower in response to the “self” voice than the “other” voice, indicative of dissimilar self–other processing. CSD exhibited a marked decrease in the “self” category from the placebo to psilocybin treatments in the right insula. Previous studies identified the insula within an extended self‐network (Modinos, Ormel, & Aleman, 2009), consistent with it being considered the convergence zone for global self‐representation, through its role in recognizing self–other boundaries, owing to its engagement in interoception, multisensory integration, and self‐processing (Tajadura‐Jiménez & Tsakiris, 2014). The additional lower CSD in the right auditory cortex for psilocybin also suggests its reduced engagement. In general, the spatial pattern of our finding coincides with the distribution of 5‐HT_2A_ and 5‐HT_1A_ receptors (the main psilocybin binding sites), which are highly present in the fronto‐medial neocortex and insular cortex neurons of the superficial and middle laminae (Stein et al., 2008; Talbot et al., 2012). Thus, psilocybin appears to alter self–other differentiation by modulating spatiotemporal activity in those structures typically involved in self‐referential processing.

### 4.4 Comparisons with other studies on altered self–other processing

Our results may be juxtaposed with previous studies on perceived self–other boundaries, investigating the effects of the neuropeptide oxytocin and mindfulness training. Oxytocin was found to blur the self–other differentiation (Pfundmair, Rimpel, Duffy, & Zwarg, 2018), an effect that was associated with reduced medial prefrontal cortex activity (Zhao, Luo, et al., 2016; Zhao, Yao, et al., 2016). Further experiments emphasized its capacity to re‐balance the self–other distinction by decreasing self‐ and increasing other‐related processing (Liu, Sheng, Woodcock, & Han, 2013). A smaller P300‐amplitude difference in response to self‐ and other‐stimuli was also found in meditators and negatively correlated with length of practice (Trautwein, Naranjo, & Schmidt, 2016). Meditation‐related neuroplasticity may lead to a de‐emphasis of the individual self in favor of more interdependent aspects of self–other identity (Neff, 2008). Self–other connectedness was suggested as a general mechanism of prosocial meditation effects (Trautwein, Naranjo, & Schmidt, 2014). Shared neural representations of self and other involving mirror neurons and action‐perception models are a proposed basis for prosocial human behavior, such as altruism or empathy (Decety & Sommerville, 2003; Lawrence et al., 2006). For example, overlapping self–other brain areas were found for empathetic experiences (Singer et al., 2004) and in extreme altruists (Brethel‐Haurwitz et al., 2017). The action of psychedelics in humans may produce these rare instances when a sense of shared awareness can transcend self–other differences. The feeling of connectedness or unity occurring under psilocybin may be interpreted as a shift in the meaning of “other” anchored in P300 neuronal processing. The transient functional modulation of brain dynamics may result in further cognitive and emotional reappraisals. Accordingly, psilocybin can induce a positive emotional processing bias by modulating the P300 amplitude (Kometer et al., 2012) and promote positive attitudes and behaviors toward self and others (Pokorny, Preller, Kraehenmann, & Vollenweider, 2016; Preller et al., 2016) that are observable even months after psilocybin intake (Griffiths et al., 2011).

Our data furthermore showed a general psilocybin effect in the N100 timeframe (which, however, did not meet the global duration statistics criterion) and its link with disembodiment. This suggests that an early sensory and more bodily anchored sense of self is also altered by psilocybin during states of perceived loosening of corporeal boundaries. A distinction between the *minimal (core) self*, an immediate subject of experience rooted in a bodily subject of action, as opposed to the *mental self*, which engages more elaborative representational aspects, has been delineated in recent conceptual and experimental frameworks (Blanke, 2012; Gallagher, 2000). Indeed, the N100 component has been associated with a basic form of self‐awareness (Hubl et al., 2014), and self‐recognition was found to require at least two components: self‐awareness of one's body and one's action (van den Bos & Jeannerod, 2002). A rich literature underlines the role of N100 in explaining the prereflective self‐awareness of action and source distinction in the context of the forward model of motor control (Blakemore, Wolpert, & Frith, 2002; Wolpert & Miall, 1996). This framework aims to both explain the origination of the basic sense of self while clarifying disturbances in self‐experience (Ford, Roach, & Mathalon, 2010). The present whole‐scalp topographic analysis did not specifically target the mechanism of corollary discharge, which reflects a local sensory‐motor interplay. Still, our study may motivate future investigations of these bodily aspects of psychedelic effects, in consideration of this and other neurophysiological mechanisms. Additionally, a single process early in the neural processing stream (e.g., N100) may further modulate later stages through co‐dependent temporal properties of the signal (Burgess, 2012; Taylor et al., 2019). The disembodiment aspect may also further modulate perception through a lessened corporeal reference and desynchronization of predictive coding (Giummarra, Gibson, Georgiou‐Karistianis, & Bradshaw, 2008; Ho, Preller, & Lenggenhager, 2020).

### 4.5 Cross‐domain relevance

The present findings are particularly relevant for social and cognitive neuroscience, biological psychiatry, and clinical applications. The psilocybin‐induced effect known as “ego‐dissolution” has been considered from various perspectives: phenomenology (Preller & Vollenweider, 2016), descriptive psychopathology (Scharfetter, 1981), as a core feature of spiritual experience (Griffiths, Richards, McCann, & Jesse, 2006), in psychodynamic terms as distortion of ego boundaries (Fischman, 1983), and in brain imaging studies (Lebedev et al., 2015). Our study extends these results by offering a spatiotemporal brain mechanism underlying this peculiar form of human cognition. Further, the study adds to the scientific understanding of “self,” “other,” and the self–other overlap, an effect that may modulate social cognition. Notably, patients in a recent clinical trial of psilocybin for therapy‐resistant depression reported a sense of connectedness that endured posttreatment (Carhart‐Harris, Erritzoe, Haijen, Kaelen, & Watts, 2018). A feeling of disconnection characterizes many psychiatric conditions, particularly depression (Karp, 2016), while connectedness was identified as a mediator of psychological well‐being (Lee, Dean, & Jung, 2008) and recovery in mental health (Leamy, Bird, Le Boutillier, Williams, & Slade, 2011). The effect may reflect a mechanism of action and become an instrument of interpersonal understanding in psychedelic‐assisted therapy, as an extension of the recent interest in psychedelics in clinical settings (Schenberg, 2018).

The concept of the self has become popular for its explanatory clinical value and as a research topic in psychiatry (Kircher & David, 2003). Maladaptive modes of self‐cognition may be encountered in autism (Lombardo et al., 2010), in major depression (Sheline et al., 2009), and as the core phenotypic marker in schizophrenia (Sass & Parnas, 2003). Treatment of these conditions may profit from a better understanding of the mechanisms underlying self‐processing. Therapeutic applications of psilocybin may utilize its effects on excessive self‐focus, ruminative brooding, sense of disconnection, and cognitive rigidity.

### 4.6 Limitations

Like most research in the area of cognitive neuroscience, this work has some limitations. We cannot fully rule out the possibility that the observed effects of psilocybin on “self”/”other” processing are driven by drug‐induced attentional deficits or task disengagement. While psilocybin can indeed modulate certain aspects of attention (Carter et al., 2005), our data reveal its differential effects on the processing of “self” and “other” as a statistically significant treatment × condition interaction. No main effect of drug on topography was found in the P300 timeframe. The differential effect of psilocybin is further supported by post hoc *t*‐maps and CSD results. Additionally, in the analysis of the behavioral task data, we also identified a significant treatment × condition interaction paralleling the EEG findings, while the drug effect was nominally nonsignificant without pitch conditions, with no concomitant increase in unsure responses. While the verbal‐auditory domain seems particularly unstable and affected in psychiatric conditions associated with alterations in self‐experience (Strik, Dierks, Hubl, & Horn, 2008), future studies should also consider other perceptual domains (e.g., visual or explicitly motor). While psilocybin may alter vocalizations, the visual evaluation of voice spectra did not suggest this possibility. It also remains unknown whether similar conclusions apply to other psychedelic‐like tryptamines. Further constraints relate to design in the context of psychoactive compounds. These include possible carry‐over effects from the crossover design and the time gap between the two sessions, as well as the efficacy of double‐blind procedures for testing substances with conspicuous effects. However, an inert placebo offered the cleanest control condition in this experiment.

### 4.7 Conclusions

Self is a pervasive experience of being consistent across time, subject of one's own actions, and distinct from others. Using a data‐driven approach, with the advantage of the high temporal resolution of EEG, we demonstrated psilocybin abolishes distinctiveness of self‐related scalp configurations via P300‐related mechanisms in association with altered activity in the supragenual cingulate cortex and insula. This study advances the current mechanistic understanding of self–other processing and the biological foundations of subjectivity. Pharmacological stimulation with the serotonergic psychedelic psilocybin offers an experimentally valid platform for perturbing and quantifying altered self‐referential processing.

## CONFLICT OF INTEREST

The authors declare no conflict of interests.

## Supporting information


**Appendix** **S1.** Supporting Information.

## Data Availability

The data that support the findings of this study are available from the authors upon reasonable request.
