# Supplementary material for: P300‐mediated modulations in self–other processing under psychedelic psilocybin are related to connectedness and changed meaning: A window into the self–other overlap
Source: Hum Brain Mapp. 2020 Aug 21;41(17):4982–96. doi: 10.1002/hbm.25174 (PMC7643385; doi:10.1002/hbm.25174)
Supplement: Supplementary file 1 — Appendix S1. Supporting Information. [file HBM-41-4982-s001.pdf]

Supplementary Material for:

P300-mediated modulations in self–other processing under psychedelic psilocybin are related to connectedness and changed meaning: a window into the self–other overlap

Lukasz Smigielski[1*], Michael Kometer[1], Milan Scheidegger[1], Cornelia Stress[1], Katrin H. Preller[1], Thomas Koenig[2], Franz X. Vollenweider[1]

[1]Neuropsychopharmacology and Brain Imaging, Department of Psychiatry, Psychotherapy and Psychosomatics, University Hospital of Psychiatry, University of Zurich, Lenggstrasse 31, CH-8032 Zurich, Switzerland.

[2]Translational Research Center, University Hospital of Psychiatry, University of Bern, Bolligenstrasse 111, CH-3000 Bern, Switzerland.

Inclusion and exclusion criteria for participation

Inclusion criteria: a) healthy male and female volunteers, 20–40 years of age; b) willing and able to give informed consent for participation; c) willing to refrain from drinking alcohol the day before the testing session, from drinking alcohol and caffeinated drinks on the testing days, and from consuming psychoactive substances 2 weeks before testing days and for the duration of the study; d) able and willing to comply with all study requirements.

Exclusion criteria: a) poor knowledge of the German language; b) a previous substantially adverse response to a hallucinogenic drug; c) participation in another study where pharmaceutical compounds will be given; d) present or antecedent psychiatric disorders in self or first-degree relatives; e) history of head trauma or fainting; f) recent cardiac or brain surgery; g) current use of medication known to affect brain function; h) concomitant therapy with potent inhibitors of cytochrome P-450 isoenzyme 3A4; i) presence of major internal or neurological disorders (including sepsis, pheochromocytoma, thyrotoxicosis, drug-induced fibrosis, familiar or basilar artery migraine); j) cardiovascular disease (hypertonia, coronary artery disease, heart insufficiency, myocardial infarction within the past 6 months, coronary spastic angina); k) peripheral vascular disease (thromboangiitis obliterans, luetic arteritis, severe arteriosclerosis, thrombophlebitis, Raynaud's disease); l) liver or renal disease; m) pregnant or breastfeeding women; n) women of childbearing potential who are not using effective, established contraception, o) hearing deficits.

Recruitment

Twenty-two right-handed participants (from the initial twenty-four; two volunteers declined before the study start), fluent in German, with normal or corrected to normal vision, were recruited through local advertisement platforms. No changes were made to the eligibility criteria, intervention, method, and planned outputs after the study beginning. For allocation of the participants (order of treatments), a computer-generated list of random numbers was used. The concealment was prepared by a physician with no further role in the study. A study nurse was responsible for the initial screening, independent internists performed the medical examination, and the research team psychologists conducted the interviews. Another physician with no further direct role in the experiments made final decisions on admission into study. All persons directly involved in the experiment, including the participants and experimentalists, remained blinded in regards to the order of treatments at each stage of the study. The number of participants were chosen based on previous EEG experiments with psilocybin (Kometer, Cahn, Andel, Carter, & Vollenweider, 2011; Kometer et al., 2012), as well as earlier studies using a similar analysis approach (Hubl et al., 2014; Manuel & Schnider, 2016). Data for analysis were anonymized according to a study ID number.

Study Flow Diagram

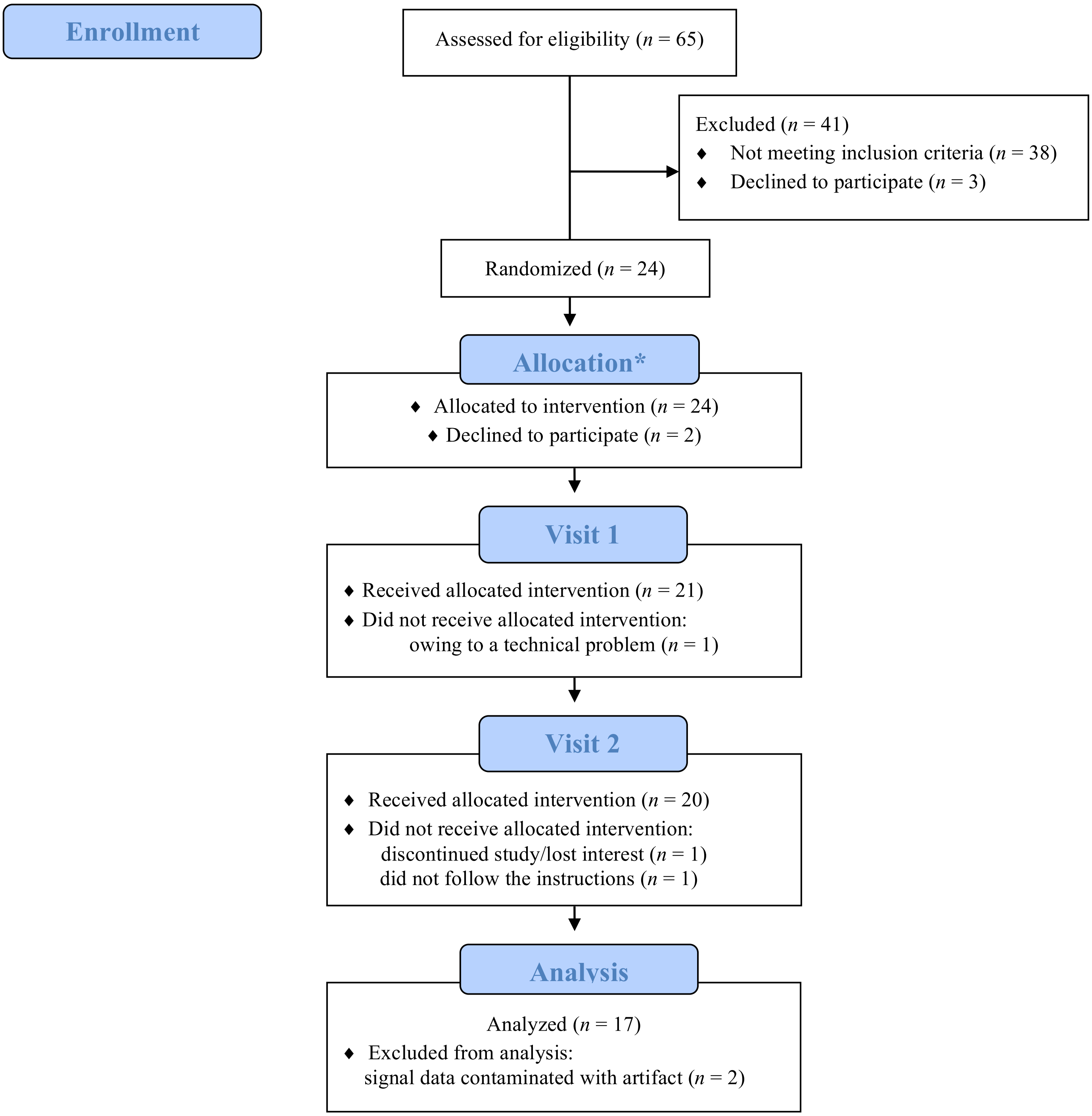

Study procedures (additional information)

Handedness was determined with the Edinburgh Handedness Inventory (Oldfield, 1971). Participants were thoroughly screened through clinical anamnesis and somatic examination, including electrocardiography and detailed blood analysis, to exclude those with serious medical conditions. The Mini-International Neuropsychiatric Interview (Sheehan et al., 1998), Symptom Checklist-90-R (Derogatis, 1996; Franke & Derogatis, 1995) and self-reported measures of substance consumption were further used to exclude subjects with present and antecedent psychiatric disorders, alcohol or drug dependence or abuse, and major psychiatric conditions in immediate relatives. To exclude major hearing deficits, the Whispered Voice Test (Macphee, Crowther, & McAlpine, 1988) was performed on each participant (all subjects scored 0, indicating good hearing). To detect possible recent use of psychoactive substances (an exclusion criterion), a urine test for amphetamine, benzodiazepines, THC, cocaine, methadone, and opiates was conducted before each experimental session. Similarly, a urine pregnancy test was conducted for female participants before substance administration.

The participants were instructed to abstain from alcoholic and caffeinated beverages in the 24 hours prior to the experiment and to eat a light breakfast on the testing day. Since psilocybin may slightly increase blood pressure, systolic and diastolic pressures were measured at regular intervals until the acute drug effects had worn off. The subjects were also supervised by a physician and released after all effects had completely subsided (7–8 hours after drug intake). Two persons were dropouts (one participated in the first, but not the second session; audio equipment problems prevented data recording for the other). Data from two other participants were excluded from analyses owing to the low quality of their EEG signals and artifact contamination. One individual did not complete the task as instructed. Six participants reported having previous occasional experiences with psilocybin or other hallucinogens. The specific moderate dose (230 μg/kg P.O.) was chosen based on previous studies in which similar doses induced significant changes in consciousness without deep thought disturbances or loss of self-control (Hasler, Grimberg, Benz, Huber, & Vollenweider, 2004; Kometer, Pokorny, Seifritz, & Volleinweider, 2015; Studerus, Kometer, Hasler, & Vollenweider, 2011). No adverse events in this study were reported. The primary outcome measures were scalp event-related potentials (ERPs) in response to the experimental stimulation (acute drug/placebo effects). The secondary measures were behavioral responses during the acute drug/placebo effects and subjective drug effects, which were assessed using the 5D-ASC rating scale. The 5D-ASC was administered 360 min after placebo/psilocybin intake as a retrospective measure of subjective effects. This is a well-validated tool for measuring etiology-independent alterations in consciousness and perception. The measure is composed of 94 visual-analogue items rated as percentage scores of an absolute scale value. The task was explained to each participant during an acclimation session at a pre-investigation visit. The study was conducted at the University Hospital of Psychiatry Zurich.

Technical details of the task

The experiment was conducted in an acoustically shielded, dimmed laboratory. The subjects were seated in front of a 16-inch computer screen and fitted with an EEG cap, microphone, and earphones. The distance between the participants' eyes and computer screen was approximately 100 cm, and that between their mouths and the microphone was fixed to about 5 cm (distances were measured at the beginning of the experiment and also controlled during the experiment). The target sound pressure level (SPL) of vocalizations, as measured at the same distance from their mouths was approximately 85 dB, and participants deviating significantly from this level were instructed to match their volume to the desired loudness at this distance. The analog audio stream consisted of a head-worn AKG C520 condenser microphone (AKG Acoustics, Vienna, Austria), low-impedance Objective2 OBJ-O2 pre-amplifier (O2, Switzerland), ER-1 insert-earphones (Etymotic Research, Elk Grove Village, IL, USA), and RME Fireface UCX audio station amplifier (RME, Haimhausen, Germany). The signal was controlled by a C++ application running on the Windows 98 operating system (Microsoft Corporation, Redmond, WA, USA) and using ASIO drivers, providing low-latency audio processing. The audio files were digitized at a sampling rate of 44.1 kHz with 16-bit depth. A digital envelope follower algorithm (which takes a high frequency signal as input and delivers the envelope of the original signal as output), was used for both "other" voice conditions to match the prerecorded file and the incoming signal in onset, offset, and amplitude. Under this procedure, the conditions were indistinguishable simply according to these three physical features. The digital processing stream of the combined setup enabled the real-time detection and modulation of participants' vocalizations with a latency as low as 5 ms as measured by a PicoScope 2000 oscilloscope (PicoTech, St. Neots, UK). Such a short delay is imperceptible (Lee, 1950; Stone & Moore, 1999) and is thus highly unlikely to influence subject performance.

References:

Derogatis, L. (1996). *SCL-90-R: Symptom Checklist-90-R: administration, scoring, and procedures manual*. Minneapolis: NCS Pearson.

Franke, G. H., & Derogatis, L. R. (1995). *Die Symptom-Checkliste von Derogatis: Manual. SCL-90-R; deutsche Version*. Göttingen: Beltz Test.

Hasler, F., Grimberg, U., Benz, M. A., Huber, T., & Vollenweider, F. X. (2004). Acute psychological and physiological effects of psilocybin in healthy humans: a double-blind, placebo-controlled dose–effect study. *Psychopharmacology, 172*(2), 145-156.

Hubl, D., Schneider, R. C., Kottlow, M., Kindler, J., Strik, W., Dierks, T., & Koenig, T. (2014). Agency and ownership are independent components of 'sensing the self' in the auditory-verbal domain. *Brain Topography, 27*(5), 672-682. doi:10.1007/s10548-014-0351-0

Kometer, M., Cahn, B. R., Andel, D., Carter, O. L., & Vollenweider, F. X. (2011). The 5-HT2A/1A agonist psilocybin disrupts modal object completion associated with visual hallucinations. *Biological Psychiatry, 69*(5), 399-406.

Kometer, M., Pokorny, T., Seifritz, E., & Volleinweider, F. X. (2015). Psilocybin-induced spiritual experiences and insightfulness are associated with synchronization of neuronal oscillations. *Psychopharmacology, 232*(19), 3663-3676. doi:10.1007/s00213-015-4026-7

Kometer, M., Schmidt, A., Bachmann, R., Studerus, E., Seifritz, E., & Vollenweider, F. X. (2012). Psilocybin biases facial recognition, goal-directed behavior, and mood state toward positive relative to negative emotions through different serotonergic subreceptors. *Biological Psychiatry, 72*(11), 898-906. doi:10.1016/j.biopsych.2012.04.005

Lee, B. (1950). Some effects of side-tone delay. *Journal of the Acoustic Society of America, 22*, 639-640.

Macphee, G. J., Crowther, J. A., & McAlpine, C. H. (1988). A simple screening test for hearing impairment in elderly patients. *Age and Ageing, 17*(5), 347-351.

Manuel, A. L., & Schnider, A. (2016). Differential processing of immediately repeated verbal and non‐verbal stimuli: an evoked‐potential study. *European Journal of Neuroscience, 43*(1), 89-97.

Oldfield, R. C. (1971). The assessment and analysis of handedness: the Edinburgh inventory. *Neuropsychologia, 9*(1), 97-113.

Sheehan, D. V., Lecrubier, Y., Sheehan, K. H., Amorim, P., Janavs, J., Weiller, E., . . . Dunbar, G. C. (1998). The Mini-International Neuropsychiatric Interview (M.I.N.I.): the development and validation of a structured diagnostic psychiatric interview for DSM-IV and ICD-10. *Journal of Clinical Psychiatry, 59 Suppl 20*, 22-33.

Stone, M. A., & Moore, B. C. (1999). Tolerable hearing aid delays. I. Estimation of limits imposed by the auditory path alone using simulated hearing losses. *Ear and Hearing, 20*(3), 182-192.

Studerus, E., Kometer, M., Hasler, F., & Vollenweider, F. X. (2011). Acute, subacute and long-term subjective effects of psilocybin in healthy humans: a pooled analysis of experimental studies. *Journal of Psychopharmacology, 25*(11), 1434-1452.
